# Surgical resection of retinoblastoma-associated bladder leiomyosarcoma during pregnancy: a case report

**DOI:** 10.1186/s12894-023-01298-3

**Published:** 2023-07-25

**Authors:** Hayato Hoshina, Satoru Taguchi, Hikaru Suyama, Kenjiro Kishitani, Yoshiyuki Akiyama, Yuta Yamada, Yusuke Sato, Daisuke Yamada, Naoya Akiba, Keiichi Kumasawa, Mayuyo Mori-Uchino, Yutaka Osuga, Haruki Kume

**Affiliations:** 1 Department of Urology, Graduate School of Medicine, The University of Tokyo, 7-3-1 Hongo, Bunkyo-Ku, Tokyo, 113-8655 Japan; 2 Department of Obstetrics and Gynecology, Graduate School of Medicine, The University of Tokyo, 7-3-1 Hongo, Bunkyo-Ku, Tokyo, 113-8655 Japan

**Keywords:** Bladder, Case report, Leiomyosarcoma, Pregnancy, Retinoblastoma

## Abstract

**Background:**

Management of a bladder tumor during pregnancy is an uncommon clinical situation. Leiomyosarcoma of the urinary bladder is a rare histological type of bladder tumor and a rare secondary cancer in survivors of retinoblastoma (RB). However, there has been no report of RB-associated bladder leiomyosarcoma during pregnancy.

**Case presentation:**

A 37-year-old pregnant woman with a medical history of RB in infancy presented with gross hematuria at the 17^th^ week of gestation. Cystoscopy revealed a 40-mm papillary tumor on the left lateral wall of the urinary bladder. At the 25^th^ week of gestation, she underwent transurethral resection of the bladder tumor, and the pathological diagnosis was bladder leiomyosarcoma with loss of *RB1* expression. At the 31^st^ week of gestation, she gave birth by caesarean section. One month after the delivery (to allow for involution of the uterus), she underwent partial cystectomy, and the specimen contained no residual leiomyosarcoma tissue.

**Conclusions:**

We have reported a case of RB-associated bladder leiomyosarcoma that was successfully treated during and after pregnancy.

**Supplementary Information:**

The online version contains supplementary material available at 10.1186/s12894-023-01298-3.

## Background

Management of a bladder tumor in a pregnant woman is a rare clinical situation. With the exception of schistosomiasis-related cases, fewer than 50 cases of bladder tumors during pregnancy have been reported in the literature to date [1–3]. Such patients warrant special attention as well as management in cooperation with obstetricians.

Leiomyosarcoma of the urinary bladder is a rare malignancy that accounts for only 0.1% of bladder lesions [4–7], and no reports to date have described treatment of this malignancy during pregnancy. Bladder leiomyosarcoma is also recognized as a rare secondary cancer in survivors of retinoblastoma (RB) [8–12]. We herein report a case of RB-associated bladder leiomyosarcoma that was successfully managed during and after pregnancy.

## Case presentation

A 37-year-old pregnant woman (gravida 2 para 1) with a medical history of bilateral RB in infancy presented with macroscopic hematuria at the 17^th^ week of gestation. At 0 years of age, she had undergone treatment for the bilateral RB by enucleation of the left eye followed by radiotherapy and photodynamic therapy to the right eye; no chemotherapy was administered. She had been proven to have a pathogenic germline variant of *RB1*, whereas she had no apparent family history of RB.

**Fig. 1:**
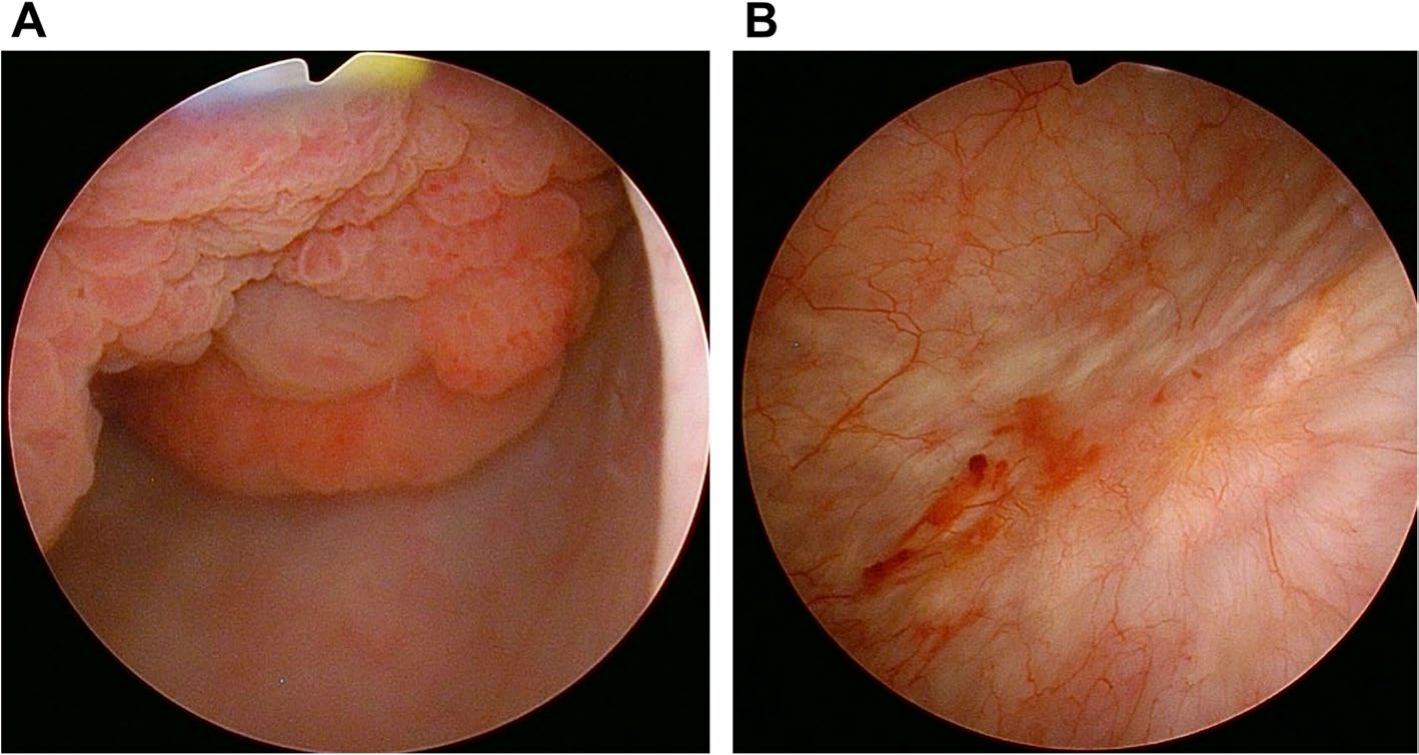
Cystoscopic findings before and after TURBT. **A** Cystoscopy before TURBT revealed an approximately 40-mm papillary tumor on the left lateral wall of the urinary bladder. **B** Cystoscopy after TURBT showed a surgical scar on the bladder lumen without obvious local recurrence. TURBT, transurethral resection of the bladder tumor

**Fig. 2:**
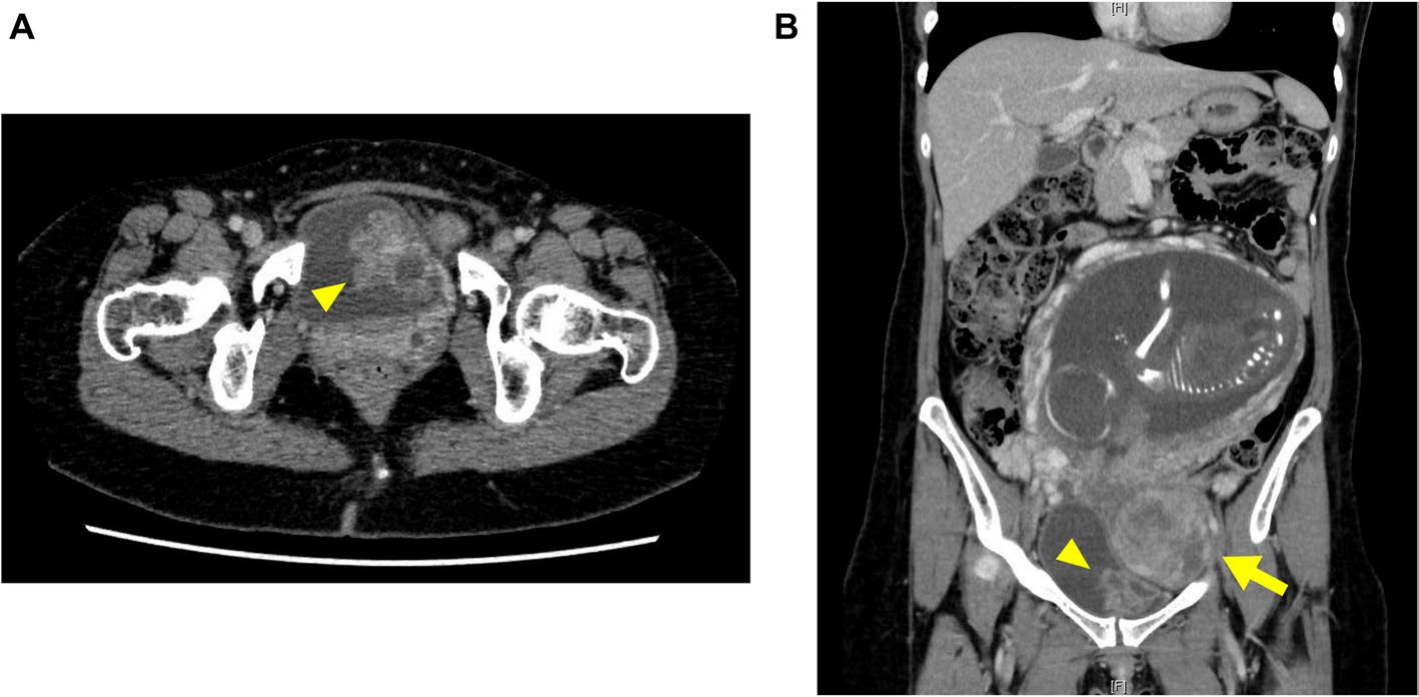
Computed tomography images at the 22^nd^ week of gestation. **A** Axial image of the tumor located on the left lateral wall of the urinary bladder (arrowhead). **B** Coronal image of the fetus, a uterine fibroid (arrow), and the bladder tumor (arrowhead)

Cystoscopy revealed an approximately 40-mm papillary tumor on the left lateral wall of the urinary bladder (Fig. 1A). The patient had class II urine cytology. After consulting obstetricians and confirming the clinical validity, she underwent contrast-enhanced computed tomography (CT) at the 22^nd^ week of gestation. CT imaging revealed the fetus, a uterine fibroid, and the above-mentioned bladder tumor without any metastatic lesions (Fig. 2A and B). Magnetic resonance imaging and ultrasonography also revealed the bladder tumor without obvious muscular invasion.

**Fig. 3:**
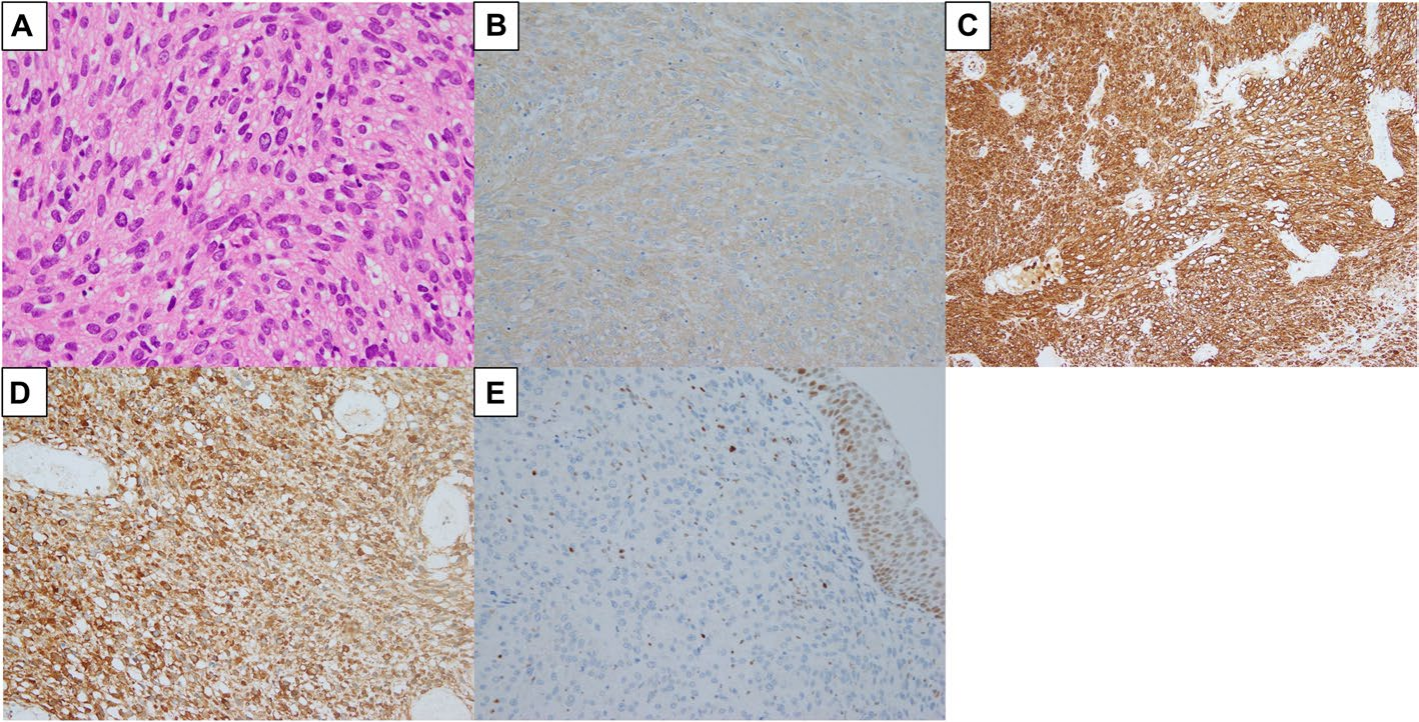
Pathological analyses of the TURBT specimen. **A** Examination revealed proliferation of spindle tumor cells in the stroma with enhanced mitotic activity (hematoxylin and eosin stain, × 400). Immunohistochemically, the tumor cells were positive for **B** smooth muscle actin (× 200), **C** caldesmon (× 200), and **D** HHF-35 (× 200). **E** The loss of *RB1* expression in the tumor cells was immunohistochemically confirmed (× 200). RB, retinoblastoma; TURBT, transurethral resection of the bladder tumor

At the 25^th^ week of gestation, the patient underwent transurethral resection of the bladder tumor (TURBT) under general anesthesia. Pathological analysis of the TURBT specimen showed stromal proliferation of spindle tumor cells with enhanced mitotic activity and an overlying benign urothelium (Fig. 3A). Immunohistochemically, the tumor cells were positive for smooth muscle actin, caldesmon, and HHF-35 and negative for other markers including p53 (Fig. 3B–D, Table S1), resulting in the pathological diagnosis of bladder leiomyosarcoma. Immunohistochemistry also demonstrated the loss of *RB1* expression in the tumor cells (Fig. 3E, Table S1), suggesting that the leiomyosarcoma might have occurred as a secondary cancer on the background of RB.

At the 31^st^ week of gestation (the timing of which was determined together with obstetricians considering both fetal intact survival and cancer treatment), the patient gave birth by caesarean section. Contrast-enhanced CT and magnetic resonance imaging after the delivery detected neither local recurrence nor metastatic lesions. Cystoscopy after TURBT and delivery showed a surgical scar on the bladder lumen without obvious local recurrence (Fig. 1B). One month after the delivery (to allow for involution of the uterus), the patient underwent partial cystectomy to assure the complete resection of the tumor. Under general anesthesia, the bladder area scheduled for resection was marked with cystoscopy in advance. Open partial cystectomy was then performed using the lower abdominal midline incision of the preceding caesarean section. The procedure was successfully completed with no intraoperative complications. Pathological assessment of the partial cystectomy specimen (35 × 22 × 7 mm) identified no residual leiomyosarcoma tissue. The patient was planned to undergo careful follow-up by both urologists and obstetricians without additional treatment. She did not experience recurrence six months after delivery and no abnormal fetal development was observed.

## Discussion and conclusions

We have described the first case of RB-associated bladder leiomyosarcoma that was successfully treated during and after pregnancy. Bladder tumors during pregnancy are very rare, and fewer than 50 cases of bladder tumors unrelated to schistosomiasis (bilharziasis) during pregnancy have been reported to date [1–3]. Although macroscopic hematuria is a major symptom of bladder tumors, it can be overlooked during pregnancy because pregnant women frequently have genitourinary bleeding from the uterus or due to cystitis. Spahn et al. [1] reviewed 27 patients with bladder cancer during pregnancy and reported that 81% of the patients presented with hematuria; however, the hematuria was initially mistaken for vaginal bleeding in 22%. Cystoscopy, ultrasonography, and urine cytology should therefore be considered for pregnant women with unclarified macroscopic hematuria. Generally, CT is not recommended for pregnant women because it exposes the fetus to radiation [3]. However, the patient in the present case underwent CT at the 22^nd^ week of gestation after consulting obstetricians and confirming its clinical validity (i.e., the organogenesis period of the fetus had already ended), and this CT examination provided essential information for subsequent treatments. TURBT under general anesthesia has been the mainstay of managing bladder tumors during malignancy [1]. At the time of the TURBT procedure in the present case, the tumor had become slightly larger than at the time of the preoperative evaluation and exhibited increased blood flow, thus necessitating careful resection. Fortunately, the uterus did not hinder the operative field as much as expected because of uterine retroflexion in the lithotomy position. The obstetricians monitored the fetal heartbeat during TURBT to ensure the safety of the fetus.

Bladder leiomyosarcoma is a rare malignancy that accounts for only 0.1% of bladder lesions [4–7]. Given the rarity of this tumor, a consensus on standard treatment pathways has not been reached [7], and no reports have described the treatment of a patient during pregnancy. Martin et al. [4] reviewed 18 patients with bladder leiomyosarcoma, 8 of whom underwent radical surgery (radical cystectomy or pelvic exenteration), 7 of whom underwent partial cystectomy, and the remaining 3 of whom underwent TURBT only. Xu et al. [5] reported that partial cystectomy, as opposed to radical cystectomy, may be a reliable option for small bladder leiomyosarcoma (< 4 cm) because it may offer similar therapeutic efficacy and better quality of life. Furthermore, Coiner et al. [6] analyzed data of 165 patients with bladder leiomyosarcoma using the Surveillance, Epidemiology, and End Results database and reported that an increased tumor size (> 5 cm) was an independent prognostic factor for the disease. Although the size of the tumor in the present case was borderline (4 cm), we considered that partial cystectomy was adequate for radical resection because the partial cystectomy specimen did not contain residual leiomyosarcoma tissue. Nevertheless, careful follow-up by both urologists and obstetricians is warranted for this case.

Bladder leiomyosarcoma is also a rare secondary cancer in survivors of RB [8–12]. The mechanisms underlying its development are not fully understood but may include the effect of the *RB1* gene itself, radiotherapy, and chemotherapy [9]. Given that our patient underwent neither chemotherapy nor radiotherapy to the urinary bladder, the development of bladder leiomyosarcoma in this case may have been primarily attributable to the effect of the *RB1* gene. Actually, the loss of *RB1* expression in the tumor cells was immunohistochemically confirmed in the TURBT specimen.

In conclusion, we have reported a case of RB-associated bladder leiomyosarcoma that was radically and safely resected during and after pregnancy. Although the management of this case was clinically challenging, close cooperation with obstetricians resulted in a successful outcome.

## Supplementary Information

**Additional file 1:** **Table S1. **Immunohistochemistry results of the TURBT specimen.

## Data Availability

The datasets used and/or analyzed during the current study are available from the corresponding author (ST) on reasonable request.

## Abbreviations

CT: Computed tomography
RB: Retinoblastoma
TURBT: Transurethral resection of the bladder tumor
