# Supplementary material for: Surgical resection of retinoblastoma-associated bladder leiomyosarcoma during pregnancy: a case report
Source: BMC Urol. 2023 Jul 25;23:125. doi: 10.1186/s12894-023-01298-3 (PMC10367244; doi:10.1186/s12894-023-01298-3)
Supplement: Supplementary file 1 — Additional file 1: Table S1. Immunohistochemistry results of the TURBT specimen. [file 12894_2023_1298_MOESM1_ESM.docx]

**Table S1.** Immunohistochemistry results of the TURBT specimen

| Stain | Result |
| --- | --- |
| SMA | (+) |
| Caldesmon | (+) |
| HHF-35 | (+) |
| Desmin | (–) |
| Calponin | (–) |
| Myogenin | (–) |
| CK AE1/AE3 | (–) |
| CK20 | (–) |
| GATA-3 | (–) |
| S-100 | (–) |
| ALK | (–) |
| MDM2 | (–) |
| p53 | (–, null) |
| RB1 | (–) |
| Ki-67 | MIB-1 index: 60–70% |

ALK, anaplastic lymphoma kinase; CK, cytokeratin; RB, retinoblastoma; SMA, smooth muscle actin; TURBT, transurethral resection of the bladder tumor; (+), positive; (–), negative
